# 3-(4-Amino-3-phenyl-5-sulfanyl­idene-4,5-dihydro-1*H*-1,2,4-triazol-1-yl)-3-(2-chloro­phen­yl)-1-phenyl­propan-1-one

**DOI:** 10.1107/S1600536811023750

**Published:** 2011-06-25

**Authors:** Yan Gao, Li-hua Zhang, He-wen Wang

**Affiliations:** a School of Chemical Engineering, University of Science and Technology LiaoNing, Anshan 114051, People’s Republic of China; b College of Chemistry and Applied Chemistry, Huanggang Normal University, Huanggang 438000, People’s Republic of China

## Abstract

In the title mol­ecule, C_23_H_19_ClN_4_OS, the 1,2,4-triazole ring forms dihedral angles of 46.5 (2), 87.4 (2) and 80.9 (2) Å with the three six-membered rings. Weak inter­molecular N—H⋯S and C—H⋯O hydrogen bonds consolidate the crystal packing.

## Related literature

For the crystal structures of related 1,2,4-triazole-5(4*H*)-thione derivatives, see: Al-Tamimi *et al.* (2010 ▶); Fun *et al.* (2009 ▶); Tan *et al.* (2010 ▶); Wang *et al.* (2011 ▶).
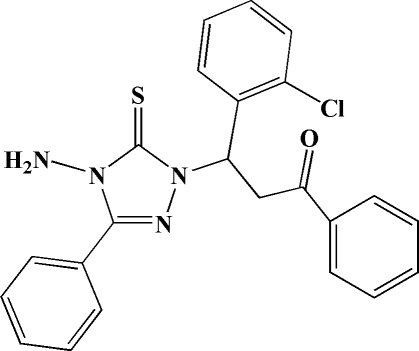


## Experimental

### 

#### Crystal data

C_23_H_19_ClN_4_OS
*M* *_r_* = 434.93
Triclinic, 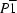

*a* = 10.559 (3) Å
*b* = 10.787 (4) Å
*c* = 10.835 (3) Å
α = 99.582 (2)°
β = 96.638 (4)°
γ = 115.267 (3)°
*V* = 1076.2 (6) Å^3^
*Z* = 2
Mo *K*α radiation
μ = 0.30 mm^−1^
*T* = 113 K
0.20 × 0.20 × 0.14 mm

#### Data collection

Rigaku Saturn CCD area-detector diffractometer
Absorption correction: multi-scan (*CrystalClear*; Rigaku/MSC, 2005 ▶) *T*_min_ = 0.943, *T*_max_ = 0.960
13931 measured reflections
5111 independent reflections
3433 reflections with *I* > 2σ(*I*)
*R*_int_ = 0.038

#### Refinement

*R*[*F* ^2^ > 2σ(*F* ^2^)] = 0.032
*wR*(*F* ^2^) = 0.079
*S* = 0.95
5111 reflections
279 parameters
H atoms treated by a mixture of independent and constrained refinement
Δρ_max_ = 0.40 e Å^−3^
Δρ_min_ = −0.29 e Å^−3^

### 

Data collection: *CrystalClear* (Rigaku/MSC, 2005 ▶); cell refinement: *CrystalClear*; data reduction: *CrystalClear*; program(s) used to solve structure: *SHELXS97* (Sheldrick, 2008 ▶); program(s) used to refine structure: *SHELXL97* (Sheldrick, 2008 ▶); molecular graphics: *SHELXTL* (Sheldrick, 2008 ▶); software used to prepare material for publication: *SHELXTL*.

## Supplementary Material

Crystal structure: contains datablock(s) global, I. DOI: 10.1107/S1600536811023750/cv5107sup1.cif

Structure factors: contains datablock(s) I. DOI: 10.1107/S1600536811023750/cv5107Isup2.hkl

Supplementary material file. DOI: 10.1107/S1600536811023750/cv5107Isup3.cml

Additional supplementary materials:  crystallographic information; 3D view; checkCIF report

## Figures and Tables

**Table 1:** Hydrogen-bond geometry (Å, °)

*D*—H⋯*A*	*D*—H	H⋯*A*	*D*⋯*A*	*D*—H⋯*A*
N4—H4*B*⋯S1^i^	0.945 (18)	2.714 (17)	3.4928 (17)	140.2 (12)
C7—H7⋯O1^ii^	0.95	2.52	3.436 (2)	161
C21—H21⋯O1^iii^	0.95	2.53	3.415 (2)	156

Symmetry codes: (i) 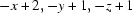; (ii) 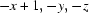; (iii) 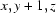.

**Fig. 1.**
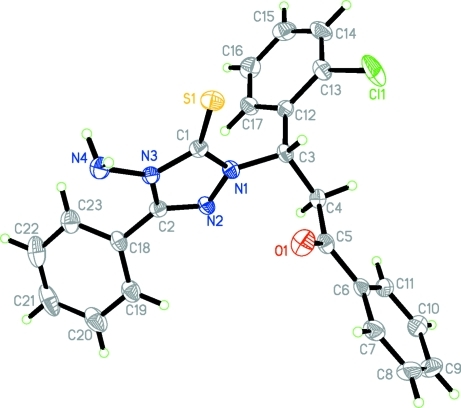
View of the molecule of (I) showing the atom-labelling scheme. Displacement ellipsoids are drawn at the 55% probability level.

## supplementary crystallographic information

### Comment

In continuation of structural study of 1,2,4-triazole-5(4*H*)-thione derivatives in our group (Wang *et al.*, 2011), we present here the crystal structure of the title compound, (I).

In (I) (Fig.1), all bond lengths and angles are normal and comparable with those observed in related structures (Al-Tamimi *et al.*, 2010; Fun *et al.*, 2009; Tan *et al.*, 2010; Wang *et al.*, 2011). The 1,2,4-triazole ring is planar with an r.m.s. deviation of 0.002 (2) Å. The C1 atom in the triazole ring deviates from the normal C*_sp_*^2^ hybridization state having the bond angles of 102.56 (10)° (N1—C1—N3) and 129.94 (10)° (N1—C1—S1). There are three benzene rings in the molecule. The three benzene rings are inclined with respect to the 1,2,4-triazole ring [dihedral angles of 46.5 (2)° (C18—C23), 87.4 (2)° (C6—C11) and 80.9 (2)% (C12—C17)]. Benzene ring A (C18—C23) attached to the triazole ring makes the dihedral angle of 90.6 (2) and 126.9 (2)° with the benzene rings B (C6—C11) and C (C12—C17), respectively. Ring B and ring C form a dihedral angle 93.6 (2)°.

In the crystal structure, weak intermolecular N—H···S and C—H···O hydrogen bonds (Table 1) consolidate the crystal packing.

### Experimental

The title compound was synthesized by the reaction of the 3-(2-chlorophenyl)-1- phenyl-2-propen-1-one (2.0 mmol) with 4-amino-3-phenyl-4*H*-1,2,4-triazole-5- thiol (2.0 mmol) in ethanol. The reaction progress was monitored *via* TLC. The resulting precipitate was filtered off, washed with cold ethanol, dried and purified to give the target product as colourless solid in 75% yield. Crystals of (I) suitable for single-crystal X-ray analysis were grown by slow evaporation of a solution in chloroform-ethanol (1:1).

### Refinement

The H atoms attached to N atoms were located in a difference map and isotropically refined. C-bound H atoms were positioned geometrically  (C—H = 0.95–1.00 Å) and refined as riding, with *U*_iso_(H) = 1.2*U*_eq_(C).

### Crystal data

**Table:**

C_23_H_19_ClN_4_OS	*Z* = 2
*M**_r_* = 434.93	*F*(000) = 452
Triclinic, *P*1	*D*_x_ = 1.342 Mg m^−^^3^
Hall symbol: -P 1	Mo *K*α radiation, λ = 0.71073 Å
*a* = 10.559 (3) Å	Cell parameters from 3478 reflections
*b* = 10.787 (4) Å	θ = 1.9–27.9°
*c* = 10.835 (3) Å	µ = 0.30 mm^−^^1^
α = 99.582 (2)°	*T* = 113 K
β = 96.638 (4)°	Prism, colourless
γ = 115.267 (3)°	0.20 × 0.20 × 0.14 mm
*V* = 1076.2 (6) Å^3^	

### Data collection

**Table:**

Rigaku Saturn CCD area-detector diffractometer	5111 independent reflections
Radiation source: rotating anode	3433 reflections with *I* > 2σ(*I*)
multilayer	*R*_int_ = 0.038
Detector resolution: 14.63 pixels mm^-1^	θ_max_ = 27.9°, θ_min_ = 2.0°
φ and ω scans	*h* = −13→13
Absorption correction: multi-scan (*CrystalClear*; Rigaku/MSC, 2005)	*k* = −14→14
*T*_min_ = 0.943, *T*_max_ = 0.960	*l* = −14→14
13931 measured reflections	

### Refinement

**Table:**

Refinement on *F*^2^	Primary atom site location: structure-invariant direct methods
Least-squares matrix: full	Secondary atom site location: difference Fourier map
*R*[*F*^2^ > 2σ(*F*^2^)] = 0.032	Hydrogen site location: inferred from neighbouring sites
*wR*(*F*^2^) = 0.079	H atoms treated by a mixture of independent and constrained refinement
*S* = 0.95	*w* = 1/[σ^2^(*F*_o_^2^) + (0.0349*P*)^2^] where *P* = (*F*_o_^2^ + 2*F*_c_^2^)/3
5111 reflections	(Δ/σ)_max_ = 0.001
279 parameters	Δρ_max_ = 0.40 e Å^−^^3^
0 restraints	Δρ_min_ = −0.29 e Å^−^^3^

### Special details

**Table:**

Geometry. All e.s.d.'s (except the e.s.d. in the dihedral angle between two l.s. planes) are estimated using the full covariance matrix. The cell e.s.d.'s are taken into account individually in the estimation of e.s.d.'s in distances, angles and torsion angles; correlations between e.s.d.'s in cell parameters are only used when they are defined by crystal symmetry. An approximate (isotropic) treatment of cell e.s.d.'s is used for estimating e.s.d.'s involving l.s. planes.
Refinement. Refinement of *F*^2^ against ALL reflections. The weighted *R*-factor *wR* and goodness of fit *S* are based on *F*^2^, conventional *R*-factors *R* are based on *F*, with *F* set to zero for negative *F*^2^. The threshold expression of *F*^2^ > σ(*F*^2^) is used only for calculating *R*-factors(gt) *etc*. and is not relevant to the choice of reflections for refinement. *R*-factors based on *F*^2^ are statistically about twice as large as those based on *F*, and *R*- factors based on ALL data will be even larger.

### Fractional atomic coordinates and isotropic or equivalent isotropic displacement parameters (Å^2^)

**Table:**

	*x*	*y*	*z*	*U*_iso_*/*U*_eq_	
S1	0.87508 (4)	0.30026 (4)	0.43912 (3)	0.02271 (9)	
Cl1	1.14768 (4)	0.15222 (4)	0.21807 (4)	0.04566 (13)	
O1	0.70047 (10)	0.13775 (10)	0.05439 (9)	0.0298 (2)	
N1	0.90922 (11)	0.39665 (11)	0.22109 (10)	0.0171 (2)	
N2	0.88351 (11)	0.48767 (11)	0.15864 (10)	0.0184 (2)	
N3	0.81314 (11)	0.49054 (11)	0.34152 (9)	0.0175 (2)	
N4	0.75491 (14)	0.52876 (15)	0.44374 (11)	0.0252 (3)	
C1	0.86846 (13)	0.39575 (13)	0.33424 (11)	0.0174 (3)	
C2	0.82420 (13)	0.54367 (13)	0.23458 (11)	0.0172 (3)	
C3	0.98141 (13)	0.31632 (13)	0.17006 (12)	0.0179 (3)	
H3	0.9499	0.2301	0.2049	0.021*	
C4	0.93753 (13)	0.26682 (13)	0.02470 (11)	0.0183 (3)	
H4C	0.9598	0.3500	−0.0121	0.022*	
H4D	0.9946	0.2203	−0.0067	0.022*	
C5	0.77970 (14)	0.16486 (13)	−0.02188 (12)	0.0201 (3)	
C6	0.72490 (13)	0.09573 (13)	−0.16117 (12)	0.0191 (3)	
C7	0.58247 (14)	−0.00620 (14)	−0.20344 (13)	0.0263 (3)	
H7	0.5211	−0.0281	−0.1443	0.032*	
C8	0.53048 (15)	−0.07543 (16)	−0.33125 (14)	0.0344 (4)	
H8	0.4331	−0.1442	−0.3600	0.041*	
C9	0.61981 (16)	−0.04485 (16)	−0.41761 (13)	0.0332 (4)	
H9	0.5838	−0.0934	−0.5053	0.040*	
C10	0.76108 (16)	0.05597 (14)	−0.37670 (13)	0.0266 (3)	
H10	0.8221	0.0770	−0.4362	0.032*	
C11	0.81364 (14)	0.12638 (13)	−0.24900 (12)	0.0214 (3)	
H11	0.9108	0.1960	−0.2210	0.026*	
C12	1.14209 (13)	0.40065 (14)	0.21643 (11)	0.0179 (3)	
C13	1.22678 (15)	0.33506 (15)	0.24249 (13)	0.0249 (3)	
C14	1.37336 (15)	0.41100 (17)	0.28825 (13)	0.0313 (4)	
H14	1.4285	0.3635	0.3066	0.038*	
C15	1.43893 (15)	0.55614 (17)	0.30705 (13)	0.0311 (3)	
H15	1.5393	0.6090	0.3392	0.037*	
C16	1.35845 (15)	0.62377 (16)	0.27904 (13)	0.0292 (3)	
H16	1.4036	0.7233	0.2899	0.035*	
C17	1.21141 (14)	0.54688 (14)	0.23495 (12)	0.0231 (3)	
H17	1.1568	0.5950	0.2170	0.028*	
C18	0.77955 (13)	0.65020 (13)	0.20813 (12)	0.0194 (3)	
C19	0.70373 (15)	0.63013 (15)	0.08661 (13)	0.0266 (3)	
H19	0.6793	0.5474	0.0224	0.032*	
C20	0.66390 (17)	0.73115 (18)	0.05937 (15)	0.0378 (4)	
H20	0.6110	0.7168	−0.0233	0.045*	
C21	0.70075 (18)	0.85272 (17)	0.15195 (16)	0.0407 (4)	
H21	0.6734	0.9217	0.1327	0.049*	
C22	0.77736 (17)	0.87375 (16)	0.27237 (16)	0.0370 (4)	
H22	0.8031	0.9577	0.3356	0.044*	
C23	0.81688 (15)	0.77314 (14)	0.30151 (14)	0.0273 (3)	
H23	0.8691	0.7877	0.3846	0.033*	
H4A	0.7078 (16)	0.4471 (16)	0.4696 (13)	0.031 (4)*	
H4B	0.8351 (18)	0.5891 (18)	0.5104 (15)	0.052 (5)*	

### Atomic displacement parameters (Å^2^)

**Table:**

	*U*^11^	*U*^22^	*U*^33^	*U*^12^	*U*^13^	*U*^23^
S1	0.02278 (18)	0.02589 (19)	0.02155 (18)	0.01071 (15)	0.00574 (14)	0.01129 (15)
Cl1	0.0438 (2)	0.0299 (2)	0.0776 (3)	0.02636 (19)	0.0153 (2)	0.0206 (2)
O1	0.0252 (5)	0.0299 (6)	0.0298 (6)	0.0068 (5)	0.0135 (5)	0.0064 (5)
N1	0.0183 (5)	0.0172 (5)	0.0188 (5)	0.0092 (5)	0.0061 (4)	0.0071 (5)
N2	0.0202 (6)	0.0184 (6)	0.0206 (6)	0.0112 (5)	0.0057 (5)	0.0073 (5)
N3	0.0153 (5)	0.0228 (6)	0.0155 (5)	0.0093 (5)	0.0052 (4)	0.0044 (5)
N4	0.0260 (7)	0.0372 (8)	0.0188 (6)	0.0181 (6)	0.0116 (5)	0.0083 (6)
C1	0.0134 (6)	0.0189 (6)	0.0179 (6)	0.0054 (5)	0.0037 (5)	0.0042 (5)
C2	0.0142 (6)	0.0194 (6)	0.0171 (6)	0.0066 (5)	0.0039 (5)	0.0047 (5)
C3	0.0205 (7)	0.0162 (6)	0.0210 (7)	0.0104 (5)	0.0068 (5)	0.0071 (5)
C4	0.0189 (7)	0.0163 (6)	0.0201 (7)	0.0077 (5)	0.0066 (5)	0.0046 (5)
C5	0.0206 (7)	0.0159 (6)	0.0262 (7)	0.0093 (6)	0.0073 (6)	0.0068 (6)
C6	0.0189 (7)	0.0143 (6)	0.0256 (7)	0.0091 (5)	0.0036 (6)	0.0056 (5)
C7	0.0190 (7)	0.0251 (8)	0.0347 (8)	0.0103 (6)	0.0047 (6)	0.0072 (6)
C8	0.0219 (8)	0.0313 (9)	0.0399 (9)	0.0085 (7)	−0.0069 (7)	0.0020 (7)
C9	0.0389 (9)	0.0312 (8)	0.0238 (8)	0.0153 (7)	−0.0066 (7)	0.0029 (7)
C10	0.0355 (8)	0.0227 (7)	0.0230 (7)	0.0131 (6)	0.0053 (6)	0.0096 (6)
C11	0.0231 (7)	0.0153 (6)	0.0247 (7)	0.0077 (6)	0.0028 (6)	0.0060 (6)
C12	0.0202 (7)	0.0224 (7)	0.0143 (6)	0.0116 (6)	0.0060 (5)	0.0053 (5)
C13	0.0285 (8)	0.0271 (8)	0.0273 (7)	0.0177 (6)	0.0100 (6)	0.0107 (6)
C14	0.0275 (8)	0.0485 (10)	0.0308 (8)	0.0271 (8)	0.0091 (7)	0.0132 (7)
C15	0.0189 (7)	0.0444 (10)	0.0271 (8)	0.0124 (7)	0.0048 (6)	0.0069 (7)
C16	0.0223 (7)	0.0271 (8)	0.0317 (8)	0.0064 (6)	0.0059 (6)	0.0037 (6)
C17	0.0212 (7)	0.0234 (7)	0.0261 (7)	0.0115 (6)	0.0042 (6)	0.0061 (6)
C18	0.0182 (6)	0.0215 (7)	0.0242 (7)	0.0108 (6)	0.0123 (6)	0.0100 (6)
C19	0.0310 (8)	0.0337 (8)	0.0255 (7)	0.0206 (7)	0.0130 (6)	0.0120 (6)
C20	0.0488 (10)	0.0559 (11)	0.0347 (9)	0.0396 (9)	0.0199 (8)	0.0254 (8)
C21	0.0553 (11)	0.0414 (10)	0.0577 (11)	0.0382 (9)	0.0363 (10)	0.0324 (9)
C22	0.0443 (10)	0.0237 (8)	0.0515 (11)	0.0181 (7)	0.0276 (9)	0.0114 (8)
C23	0.0256 (8)	0.0253 (8)	0.0320 (8)	0.0111 (6)	0.0122 (6)	0.0069 (6)

### Geometric parameters (Å, °)

**Table:**

S1—C1	1.6705 (13)	C9—C10	1.381 (2)
Cl1—C13	1.7397 (16)	C9—H9	0.9500
O1—C5	1.2247 (14)	C10—C11	1.3828 (18)
N1—C1	1.3456 (15)	C10—H10	0.9500
N1—N2	1.3791 (13)	C11—H11	0.9500
N1—C3	1.4628 (15)	C12—C13	1.3886 (17)
N2—C2	1.3080 (15)	C12—C17	1.3926 (18)
N3—C2	1.3712 (16)	C13—C14	1.3847 (19)
N3—C1	1.3736 (16)	C14—C15	1.381 (2)
N3—N4	1.4130 (14)	C14—H14	0.9500
N4—H4A	0.918 (15)	C15—C16	1.375 (2)
N4—H4B	0.945 (18)	C15—H15	0.9500
C2—C18	1.4716 (17)	C16—C17	1.3871 (18)
C3—C12	1.5138 (17)	C16—H16	0.9500
C3—C4	1.5233 (17)	C17—H17	0.9500
C3—H3	1.0000	C18—C19	1.3910 (18)
C4—C5	1.5158 (17)	C18—C23	1.3974 (18)
C4—H4C	0.9900	C19—C20	1.3861 (19)
C4—H4D	0.9900	C19—H19	0.9500
C5—C6	1.4913 (18)	C20—C21	1.383 (2)
C6—C11	1.3926 (17)	C20—H20	0.9500
C6—C7	1.3935 (18)	C21—C22	1.382 (2)
C7—C8	1.3809 (19)	C21—H21	0.9500
C7—H7	0.9500	C22—C23	1.3865 (19)
C8—C9	1.384 (2)	C22—H22	0.9500
C8—H8	0.9500	C23—H23	0.9500
			
C1—N1—N2	113.49 (10)	C9—C10—C11	119.92 (13)
C1—N1—C3	124.44 (10)	C9—C10—H10	120.0
N2—N1—C3	121.98 (10)	C11—C10—H10	120.0
C2—N2—N1	104.42 (10)	C10—C11—C6	120.33 (13)
C2—N3—C1	109.50 (10)	C10—C11—H11	119.8
C2—N3—N4	125.04 (11)	C6—C11—H11	119.8
C1—N3—N4	125.45 (10)	C13—C12—C17	117.10 (12)
N3—N4—H4A	105.0 (9)	C13—C12—C3	121.22 (12)
N3—N4—H4B	104.9 (9)	C17—C12—C3	121.68 (11)
H4A—N4—H4B	106.4 (13)	C14—C13—C12	121.95 (13)
N1—C1—N3	102.56 (10)	C14—C13—Cl1	118.52 (11)
N1—C1—S1	129.94 (10)	C12—C13—Cl1	119.53 (11)
N3—C1—S1	127.46 (9)	C15—C14—C13	119.62 (13)
N2—C2—N3	110.02 (11)	C15—C14—H14	120.2
N2—C2—C18	124.06 (11)	C13—C14—H14	120.2
N3—C2—C18	125.91 (10)	C16—C15—C14	119.78 (14)
N1—C3—C12	110.60 (10)	C16—C15—H15	120.1
N1—C3—C4	111.47 (10)	C14—C15—H15	120.1
C12—C3—C4	112.37 (10)	C15—C16—C17	120.11 (14)
N1—C3—H3	107.4	C15—C16—H16	119.9
C12—C3—H3	107.4	C17—C16—H16	119.9
C4—C3—H3	107.4	C16—C17—C12	121.41 (12)
C5—C4—C3	112.76 (10)	C16—C17—H17	119.3
C5—C4—H4C	109.0	C12—C17—H17	119.3
C3—C4—H4C	109.0	C19—C18—C23	119.75 (13)
C5—C4—H4D	109.0	C19—C18—C2	119.19 (12)
C3—C4—H4D	109.0	C23—C18—C2	121.02 (12)
H4C—C4—H4D	107.8	C20—C19—C18	119.86 (14)
O1—C5—C6	121.17 (12)	C20—C19—H19	120.1
O1—C5—C4	120.25 (12)	C18—C19—H19	120.1
C6—C5—C4	118.56 (10)	C21—C20—C19	120.33 (15)
C11—C6—C7	119.30 (12)	C21—C20—H20	119.8
C11—C6—C5	121.73 (12)	C19—C20—H20	119.8
C7—C6—C5	118.92 (11)	C22—C21—C20	120.00 (14)
C8—C7—C6	120.06 (13)	C22—C21—H21	120.0
C8—C7—H7	120.0	C20—C21—H21	120.0
C6—C7—H7	120.0	C21—C22—C23	120.39 (15)
C7—C8—C9	120.19 (14)	C21—C22—H22	119.8
C7—C8—H8	119.9	C23—C22—H22	119.8
C9—C8—H8	119.9	C22—C23—C18	119.67 (14)
C10—C9—C8	120.20 (14)	C22—C23—H23	120.2
C10—C9—H9	119.9	C18—C23—H23	120.2
C8—C9—H9	119.9		
			
C1—N1—N2—C2	−0.53 (14)	C8—C9—C10—C11	0.2 (2)
C3—N1—N2—C2	−177.29 (11)	C9—C10—C11—C6	0.3 (2)
N2—N1—C1—N3	0.61 (13)	C7—C6—C11—C10	−0.26 (19)
C3—N1—C1—N3	177.27 (11)	C5—C6—C11—C10	177.17 (11)
N2—N1—C1—S1	178.26 (9)	N1—C3—C12—C13	145.32 (12)
C3—N1—C1—S1	−5.07 (19)	C4—C3—C12—C13	−89.41 (14)
C2—N3—C1—N1	−0.45 (13)	N1—C3—C12—C17	−34.62 (16)
N4—N3—C1—N1	−179.92 (11)	C4—C3—C12—C17	90.66 (14)
C2—N3—C1—S1	−178.18 (10)	C17—C12—C13—C14	1.71 (19)
N4—N3—C1—S1	2.35 (19)	C3—C12—C13—C14	−178.22 (12)
N1—N2—C2—N3	0.21 (13)	C17—C12—C13—Cl1	−178.59 (9)
N1—N2—C2—C18	179.04 (11)	C3—C12—C13—Cl1	1.47 (17)
C1—N3—C2—N2	0.15 (14)	C12—C13—C14—C15	−1.0 (2)
N4—N3—C2—N2	179.62 (11)	Cl1—C13—C14—C15	179.27 (10)
C1—N3—C2—C18	−178.65 (12)	C13—C14—C15—C16	−0.6 (2)
N4—N3—C2—C18	0.8 (2)	C14—C15—C16—C17	1.5 (2)
C1—N1—C3—C12	−87.95 (14)	C15—C16—C17—C12	−0.8 (2)
N2—N1—C3—C12	88.45 (13)	C13—C12—C17—C16	−0.78 (19)
C1—N1—C3—C4	146.26 (11)	C3—C12—C17—C16	179.15 (11)
N2—N1—C3—C4	−37.34 (15)	N2—C2—C18—C19	45.75 (18)
N1—C3—C4—C5	−63.58 (13)	N3—C2—C18—C19	−135.61 (13)
C12—C3—C4—C5	171.62 (10)	N2—C2—C18—C23	−131.78 (13)
C3—C4—C5—O1	4.68 (17)	N3—C2—C18—C23	46.86 (18)
C3—C4—C5—C6	−173.49 (10)	C23—C18—C19—C20	−0.91 (19)
O1—C5—C6—C11	179.54 (12)	C2—C18—C19—C20	−178.47 (12)
C4—C5—C6—C11	−2.31 (17)	C18—C19—C20—C21	0.8 (2)
O1—C5—C6—C7	−3.03 (18)	C19—C20—C21—C22	−0.1 (2)
C4—C5—C6—C7	175.12 (11)	C20—C21—C22—C23	−0.4 (2)
C11—C6—C7—C8	−0.16 (19)	C21—C22—C23—C18	0.4 (2)
C5—C6—C7—C8	−177.66 (12)	C19—C18—C23—C22	0.32 (19)
C6—C7—C8—C9	0.6 (2)	C2—C18—C23—C22	177.84 (11)
C7—C8—C9—C10	−0.6 (2)		

### Hydrogen-bond geometry (Å, °)

**Table:**

*D*—H···*A*	*D*—H	H···*A*	*D*···*A*	*D*—H···*A*
N4—H4B···S1^i^	0.945 (18)	2.714 (17)	3.4928 (17)	140.2 (12)
C7—H7···O1^ii^	0.95	2.52	3.436 (2)	161
C21—H21···O1^iii^	0.95	2.53	3.415 (2)	156

Symmetry codes: (i) −*x*+2, −*y*+1, −*z*+1; (ii) −*x*+1, −*y*, −*z*; (iii) *x*, *y*+1, *z*.
